# Local diagnostic reference levels for pediatric fluoroscopy in a Brazilian tertiary referral center: A weight‑based analysis

**DOI:** 10.1002/acm2.70508

**Published:** 2026-03-15

**Authors:** Claudia M. C. Ribeiro, Tainá O. Chaves, Marcos Decnop, Renato Gonçalves de Mendonça, Margareth Catoia Varela, Cesar Augusto Viana de Araujo, Saint Clair Gomes Junior

**Affiliations:** ^1^ Department of Radiology, Fernandes Figueira National Institute of Women's Children's and Adolescents’ Health (IFF/FIOCRUZ) Rio de Janeiro Rio de Janeiro Brazil; ^2^ Department of Radiology National Cancer Institute (INCa) Rio de Janeiro Rio de Janeiro Brazil; ^3^ Evandro Chagas National Institute of Infectious Diseases (INI/FIOCRUZ) Rio de Janeiro Rio de Janeiro Brazil; ^4^ Research Coordination Service, Fernandes Figueira National Institute of Women's Children's and Adolescents’ Health (IFF/FIOCRUZ) Rio de Janeiro Rio de Janeiro Brazil

**Keywords:** Diagnostic reference levels, pediatric diagnostic fluoroscopy, Radiation protection

## Abstract

**Purpose:**

To establish the first DRLs for contrast‐enhanced diagnostic pediatric fluoroscopic procedures in Brazil, stratified by body weight, based on data collected in a single tertiary referral public hospital of Rio de Janeiro city.

**Methods:**

This descriptive, cross‐sectional study included 928 diagnostic fluoroscopy examinations performed in patients aged 0–18 years between December 2020 and December 2024 at a national pediatric referral center. Dosimetric parameters such as Air kerma‐area product (PKA), Air kerma at patient entrance reference point (Ka,r), fluoroscopy time, and number of images were extracted from automatically generated reports. Results were compared with existing international DRLs.

**Results:**

Local median doses showed overall agreement with European and UK reference levels in lower weight and age groups, but higher values were observed in patients with higher body mass. Compared with other single‐center studies, local KAP values were higher, likely reflecting differences in case complexity and exposure parameters. Weight‐based stratification proved more reliable than age‐based grouping for defining pediatric DRLs, supporting its use as the preferred reference criterion.

**Conclusion:**

This is the first Brazilian study to systematically report DRLs for diagnostic pediatric fluoroscopy using weight‐based stratification, that support the need for structured dose monitoring, protocol optimization, and continuous professional training.

## 1 INTRODUCTION

The use of Diagnostic Reference Levels (DRLs) has been increasingly incorporated into national regulations. In Brazil, Resolution RDC No. 611/2022 of the National Health Surveillance Agency (ANVISA) recommends the implementation of DRLs as part of optimization programs for medical exposures in both adults and children.^1^

Aligned with this movement, the Brazilian College of Radiology and Diagnostic Imaging (CBR) launched, in 2022, the NRD Brasil and NRD Latin campaigns, aiming to foster the collection of national and South American data and promote a culture of dose optimization.^2^, ^3^

DRLs recommended by national and international institutions are fundamental tools for the standardization and optimization of practices in diagnostic and interventional radiology.^4^, ^5^, ^6^ According to the International Commission on Radiological Protection (ICRP), DRLs are defined as levels of radiation dose metrics for typical examinations in standardized patient groups or standard phantoms, obtained and collected locally, nationally, or regionally, with the aim of identifying practices that involve doses significantly above expected levels.^7^, ^8^

Although less frequently used today due to the widespread availability of tomographic and magnetic resonance techniques, diagnostic fluoroscopy remains essential in pediatric practice for the functional evaluation of the urinary and gastrointestinal systems.^9^, ^10^ This method involves the use of real‐time ionizing radiation, which can result in high doses if technical parameters are not strictly controlled.^10^, ^11^, ^12^, ^13^ This concern is particularly relevant in pediatrics, as children are significantly more susceptible to the stochastic effects of radiation than adults, with radiosensitivity that may be 2–10 times higher.^13^, ^14^ Factors contributing to this increased risk include higher mitotic activity, longer life expectancy for the manifestation of damage, a greater proportion of radiosensitive tissues, and anatomical characteristics that favor radiation scattering.^15^, ^16^

The heterogeneity in technical protocols used for pediatric diagnostic fluoroscopy, combined with the scarcity of local dose data, may lead to unnecessarily high exposures.^5^, ^14^, ^17^, ^18^ Since 2013, the European Commission (EC) has mandated the use of DRLs in member countries^4^, ^6^; however, only about 20% of European countries have their own dose data for pediatric fluoroscopic studies.^4^, ^8^ With a focus on practical guideline development, campaigns such as Pause and Pulse have proposed strategies to rationalize the use of fluoroscopy in children, including recommendations on image acquisition, collimation, and exposure time.^9^, ^19^

The establishment of pediatric DRLs should be guided by the concept of population collective dose—meaning the inclusion of both frequent low‐dose exams and rare exams with potentially high doses.^7^, ^8^ The ICRP itself acknowledges that in contexts with limited case numbers, especially in pediatrics, initial DRLs may be derived from small samples, with updates planned as additional data become available.^7^ Stratification by body weight, rather than age, is particularly recommended by the EC to reduce intragroup anthropometric variability, thereby increasing the accuracy of estimated dosimetric values.^8^

In Brazil, consolidated DRL values for pediatric fluoroscopic examinations— especially when stratified by body weight—are not yet available. This hampers international comparisons and the development of optimization policies based on local data.

This study was conducted at a public tertiary hospital affiliated with the Unified Health System (SUS), which serves as a national reference center for rare diseases and is one of the few Brazilian institutions with sufficient expertise and volume in contrast‐enhanced pediatric fluoroscopy. The aim of this study was to establish DRLs for contrast‐enhanced pediatric fluoroscopic examinations, stratified by weight, based on data from a tertiary referral center.

## 2 MATERIALS AND METHODS

### 2.1 Study design, population, and setting

This was a descriptive, cross‐sectional study of diagnostic fluoroscopy examinations performed between December 2020 and December 2024.

The study population included patients aged 0–18 years with a clinical indication for such procedures.

### 2.2 Context

The National Institute for Women's, Children's and Adolescents’ Health Fernandes Figueira (IFF) is a public tertiary health institution affiliated with the Oswaldo Cruz Foundation (FIOCRUZ) and a national referral center for the diagnosis and treatment of genetic diseases and congenital malformations. It is certified by the Brazilian Ministry of Health as a reference service for rare diseases. Affiliated with the Unified Health System (SUS), the institution provides highly specialized care to pediatric patients with complex clinical conditions, supported by a qualified multidisciplinary team and advanced diagnostic infrastructure.

All fluoroscopic examinations were performed by board‐certified radiologists with a minimum of five years of experience in pediatric radiology. Procedures were conducted in a dedicated fluoroscopy room using a digital remote‐controlled fluoroscopy system (Siemens Luminos dRF Max), with protocols adapted for the pediatric population based on the Pause and Pulse campaign and international best practice guidelines.

### 2.3 Technical protocols and clinical conduct

Protocols were individually selected by pediatric radiologists according to study type, and included several dose optimizations tools, such as maximum collimation, restricted use of electronic magnification, because it contributes to unnecessary radiation exposure. In this system, a value of 0 indicates that no electronic magnification was applied, while values from 1 to 3 correspond to progressive levels of magnification, each associated with a proportional increase in patient radiation dose; pulse rate reduction, as low as 3 or 7,5 pulses per second; additional copper filtration, automatic exposure control, and the use of last image hold for voiding micturating cystourethrograms (MCU).

Radiologists were allowed to adjust technical parameters autonomously, when necessary, to ensure diagnostic accuracy—even if such adjustments led to increased radiation dose.

Routine quality control tests were performed on the equipment by the institution's Medical Physics team, in accordance with institutional protocols and the recommendations of the Brazilian Health Regulatory Agency (ANVISA) Resolution RDC No. 611/2022.^1^ The accuracy of the system was verified using a VacuDAP meter (VacuTec Messtechnik GmbH, serial number 1500556), with a valid calibration certificate, and demonstrated an accuracy of approximately ± 5% for pediatric fluoroscopy and approximately ± 10% for pediatric radiography.

### 2.4 Inclusion and exclusion criteria

Inclusion criteria: all fluoroscopy examinations performed in patients aged 018 years between December 2020 and December 2024.

Exclusion criteria: examinations lacking information on weight or dose, and records in which images from more than one patient were mistakenly saved under the same study.

Although ICRP recommendations establish a minimum of 20 patients per stratum to ensure statistical validity in defining DRLs, this criterion was not adopted as an exclusion factor in the present study. This decision is justified by the specialized nature of the participating center and the low prevalence of certain examinations in the pediatric population. According to ICRP Publication 135 (2017),^7^ the process of establishing and updating DRLs should be flexible and dynamic, particularly in contexts where data are limited or originate from few centers. Therefore, the initial definition of DRLs based on such data is accepted while broader surveys are awaited.

### 2.5 Data sources and variables

A standardized form was created to extract clinical and demographic data from the patients, including: sex, date of birth, date of examination, weight/mass, height, clinical indication, exam type, performing physician, and referring service.

Dosimetric data were extracted from automatically generated reports at the end of each examination, including the following parameters: PKA (µGy·m^2^), Ka,r (mGy), total number of images acquired, total fluoroscopy time (minutes), and magnification level used.

Air kerma at patient entrance reference point (Ka,r) is the total amount of radiation energy transferred to the air at the point where the X‐ray beam enters the patient, accumulated throughout the entire procedure.^7^

Air kerma‐area product (PKA) represents the product of the air kerma and the irradiated beam area, providing an estimate of the total amount of energy emitted, taking into account both the intensity and the field size,^7^ and the values were converted from microgray‐square meters (µGy·m^2^) to milligray‐square centimeters (mGy·cm^2^) using the following equivalence:

1 µGy·m^2^ = 10 mGy·cm^2^, as per ICRP^7^ and EC^8^ guidelines.

### 2.6 Stratification by age and weight

Age was calculated based on the date of birth and categorized into age groups recommended by the ICRP^7^ and EC^8^: <1, 1–5, 5–10, 10–15, and >15 years.

Weight/mass was stratified according to ICRP^7^ and EC^8^ recommendations: <5, 5 to <15, 15 to <30, 30 to <50, and 50 to <80 kg.

To facilitate comparative analysis and visualization of results, considering that most studies in the literature are based on age, these weight categories were converted into equivalent age groups (0, 1, 5, 10, and 15 years), based on the weight–age correspondence table proposed by international guidelines (Table 1).

**TABLE 1:** Weight‐based stratification for pediatric DRLs recommended by European guidelines, with equivalent ages and weight/age groups used in previous publications.

Description	Weight group (kg)	Age group based on weight‐for age charts	Most common weight groups used for the previous DRLs	Most common age groups used for the previous national DRLs (years)
Neonate	<5	<1 month	5	0
Infant, toddler and early childhood	5 to <15	1 month to <4 years	15	1
Middle childhood	15 to <30	4 to <10 years	30	5
Early adolescence	30 to <50	10 to <14 years	50	10
Late adolescence	50 to <80	14 to <18 years	80	15

### 2.7 Statistical methods

Data were entered using REDCap, and statistical analysis was performed using the JASP software package, version 0.19.3. (JASP Team, University of Amsterdam, Amsterdam, The Netherlands). Numerical data were described using the median and interquartile range (IQR), and categorical data using absolute and relative frequencies. DRL calculation followed ICRP^7^ and EC^8^ recommendations, using dose quartiles for each weight stratum observed in the selected fluoroscopic exams. PKA was considered the primary dosimetric parameter, with the 25th percentile (P25), median (P50), and 75th percentile (P75) values obtained. In addition, median values of secondary indicators were analyzed, including fluoroscopy time (minutes), Ka,r (mGy), and number of images.

## 3 RESULTS

A total of 938 diagnostic fluoroscopy examinations were performed between December 2020 and December 2024. Of these, 10 examinations were excluded from the analysis: 5 due to missing dosimetric or anthropometric data, 3 due to the inclusion of images from more than one patient in the same study, and 2 that did not correspond to contrast‐enhanced diagnostic exams (they assessed diaphragmatic mobility and central venous catheter positioning). The final sample included 928 valid diagnostic examinations (Table 2).

**TABLE 2:** Distribution of demographic (sex and age), anthropometric (weight [kg], height[m], and BMI [body mass index], clinical indication (ICD—International classification of diseases), and examination characteristics (FT [fluoroscopy time], number of images, PKA [Air kerma‐area product], Ka,r [Air kerma at patient entrance reference point]) and exam type: BE (barium enema), MS (modified barium swallow), BS (barium swallow), FG (fluoroscopy genitography). BM (barium meal), SBFT (small bowel follow‐through), MCU (micturating cystourethrograms), IVU (intravenous urography).

	*n*	%	Median	(Q1–Q3)
Sex				
Female	370	39.9		
Male	557	60.1		
Age	928	0.0	2	(0.0–7.0)
Age group				
1 (<1 year)	339	36.7		
1 (1 to <5 years)	274	29.5		
5 (5 to <10 y*ears*)	154	16.5		
10 (10 to <15 y*ears*)	107	11.5		
15 (>15 y*ears*)	54	5.8		
Weight kg	928		11.5	(5.1–22.4)
Weight group				
0 (<5 kg)	226	24.3		
5 (5 to <15 kg)	341	36.8		
15 (15 to <30 kg)	195	21.0		
30 (30 to <50 kg)	115	12.4		
50 (50 to <80 kg)	51	5.5		
Height m	841	90.6	0.9	(0.6–1.2)
BMI	841	90.6	15.7	(13.8–18.2)
ICD D,J,P,S,Z	31	3.3		
K	218	23.5		
N	225	24.2		
Q	362	39.0		
R	92	9.9		
Exam				
BE	180	19.4		
MS	51	5.5		
BS	118	12.7		
FG	16	1.7		
BM	216	23.3		
SBFT	44	4.7		
MCU	302	32.5		
IVU	1	0.1		
Number of images	909	97.9	338	(121.0–667.0)
FT	928	100.0	161	(97.5–247.5)
PKA	928	100.0	712.2	(297.5–1758.9)
Ka,r	928	100.0	2,8	(1.5–6.0)

Age was calculated based on the date of birth and categorized into age groups recommended by the ICRP^7^ and EC^8^: <1 year (0), 1 to <5 years (1), 5 to <10 years (5), 10 to <15 years (10), and >15 years (15). Weight/mass was stratified according to ICRP^7^ and EC^8^ recommendations: <5, 5 to <15, 15 to <30, 30 to <50, and 50 to <80 kg.

### 3.1 Demographic and clinical characteristics

There was a predominance of male patients (60.1%), children aged up to 5 years (66.2%), and children weighing less than 15 kg (61.1%).

The most frequent clinical indications, grouped by ICD‐10 system classification, were: congenital malformations (39.0%), diseases of the genitourinary system (24.2%), and diseases of the digestive system (23.5%).

### 3.2 Distribution by type of examination

**FIGURE 1:**
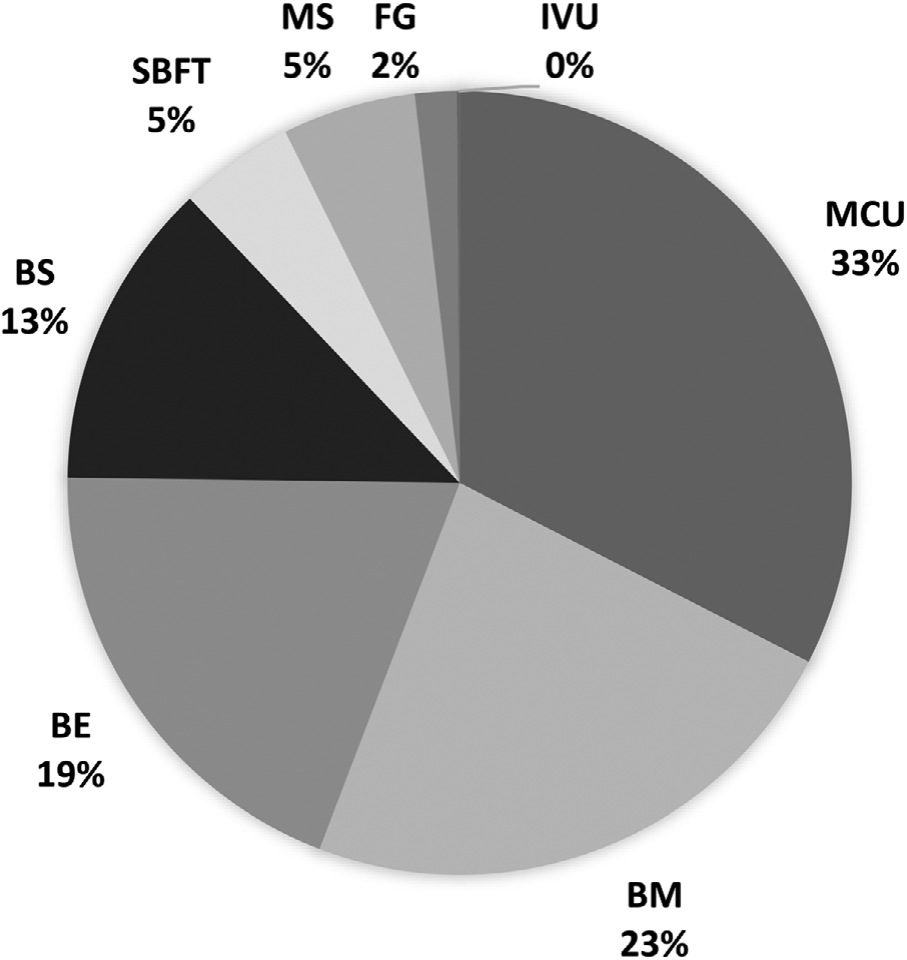
Frequency distribution of valid fluoroscopic examinations. MCU (micturating cystourethrograms), BM (barium meal), BE (barium enema), BS (barium swallow), MS (modified barium swallow), SBFT (small bowel follow‐through), FG (fluoroscopy genitography) and IVU (intravenous urography).

Figure 1 shows the relative frequency of the types of examinations performed. The four most common procedures were: MCU (33%), barium meal (BM) (23%), barium enema (BE) (19%), and Barium Swallow (BS) (13%).

Other examinations included: small bowel follow‐through (SBFT) (5%), Modified Swallow (MS) (5%), Fluoroscopy Genitography (FG) (2%), and Intravenous Urography (IVU) (<1%).

For standardization purposes, all studies involving iodinated or barium contrast administered rectally or via colostomy for colon evaluation were classified as contrast enema.

### 3.3 Dosimetric distribution by age

The dose table by age group (Table 3) was included due to data availability and the need to maintain comparability with previous studies. Although age does not accurately represent the actual distribution of radiation doses, its use was retained to facilitate comparative analysis and discussion of the results in accordance with consolidated international literature, which traditionally employs conversion tables as a stratification parameter rather than actual body mass values.

**TABLE 3:** Distribution of number of images, fluoroscopy time (min), PKA (Air kerma‐area product ‐mGy·m^2^), Ka,r (Air kerma at patient entrance reference point—mGy), according to age category. Q1–Q3 (interquartile interval). MCU (micturating cystourethrograms), BM (barium meal), BE (barium enema), BS (barium swallow), MS (modified barium swallow), SBFT (small bowel follow‐through), FG (fluoroscopy genitography).

	N images				FT				PKA				Ka,r			
	*N*	Median	(Q1–Q3)	*p* value	*N*	Median	(Q1–Q3)	*p* value	*N*	Median	(Q1–Q3)	*p* value	*N*	Median	(Q1–Q3)	*p* value
**BE**																
0	69	163	(77–431)	0.497	69	2	(1–3)	0.424	69	307	(192–580)	0.000	69	2	(1–4)	0.000
1	57	136	(52–303)		58	2	(1–3)		58	714	(370–1.390)		58	3	(1–6)	
5	25	140	(44–219)		27	2	(1–3)		27	1698	(617–2992)		27	5	(3–7)	
10	19	165	(16–310)		19	2	(1–3)		19	2859	(1840–5604)		19	6	(3–12)	
15	7	231	(10–247)		7	3	(2–6)		7	11472	(5405–17281)		7	22	(13–32)	
**MS**																
0	15	812	(393–1.300)	0.273	16	3	(2–4)	0.306	16	343	(177–761)	0.006	16	2	(1–4)	0.030
1	22	885	(513–1.431)		23	4	(2–6)		23	797	(393–1.057)		23	2	(2–4)	
5	7	641	(411–1.001)		8	2	(2–5)		8	542	(397–812)		8	2	(1–2)	
10	2	797	(322–1.272)		2	3	(2–4)		2	2757	(1761–3752)		2	6	(4–7)	
15	2	2261	(1753–2768)		2	7	(6–8)		2	3685	(3331–4039)		2	8	(8–9)	
**BS**																
0	40	410	(152–820)	0.529	40	2	(1–3)	0.987	40	241	(70–364)	0.000	40	1	(0–3)	0.008
1	19	483	(360–804)		21	2	(1–3)		21	406	(253–779)		21	2	(1–3)	
5	31	418	(118–953)		32	2	(1–3)		32	683	(360–1219)		32	2	(1–3)	
10	16	504	(299–651)		16	2	(2–3)		16	930	(682–1815)		16	2	(2–4)	
15	8	297	(208–537)		9	2	(1–3)		9	1318	(1140–2539)		9	4	(2–5)	
**FG**																
0	9	694	(544–1054)	0.678	9	4	(3–4)	0.461	9	486	(358–1839)	0.162	9	4	(3–9)	0.118
1	6	1061	(660–1400)		6	4	(3–5)		6	1529	(1150–1706)		6	9	(8–9)	
5	1	673	(673–673)		1	7	(7–7)		1	22535	(22535–22535)		1	64	(64–64)	
**BM**																
0	79	518	(155–1086)	0.189	79	3	(2–5)	0.240	79	384	(187–679)	0.000	79	2	(2–4)	0.000
1	77	357	(94–816)		77	4	(2–6)		77	807	(519–1392)		77	3	(2–6)	
5	32	324	(104–565)		34	3	(1–5)		34	992	(586–2291)		34	4	(2–8)	
10	15	544	(247–786)		15	4	(2–7)		15	2789	(813–6539)		15	7	(2–12)	
15	11	717	(19–1521)		11	3	(3–5)		11	4250	(2167–7949)		11	8	(6–14)	
**SBFT**																
0	30	202	(92–557)	0.532	32	3	(2–5)	0.439	32	410	(206–780)	0.002	32	2	(2–5)	0.037
1	8	415	(61–943)		8	5	(1–6)		8	2305	(854–2447)		8	6	(3–9)	
5	1	125	(125–125)		1	1	(1–1)		1	452	(452–452)		1	1	(1–1)	
10	3	987	(157–1993)		3	3	(2–11)		3	4475	(2314–24827)		3	8	(5–55)	
**MCU**																
0	89	412	(255–659)	0.000	93	3	(2–5)	0.000	93	210	(139–427)	0.000	93	2	(1–4)	0.000
1	81	393	(166–728)		81	3	(2–5)		81	871	(488–1557)		81	3	(2–6)	
5	51	317	(137–584)		52	3	(2–4)		52	1330	(638–3813)		52	4	(2–9)	
10	51	215	(79–387)		51	2	(2–3)		51	2587	(1391–6388)		51	5	(3–10)	
15	25	170	(99–268)		25	1	(1–2)		25	2080	(989–3582)		25	4	(2–6)	
**IVU**																
0	1	141	(141–141)	—	1	1	(1–1)	—	1	1405	(1405–1405)	—	1	5	(5–5)	—

### 3.4 Dosimetric distribution by weight

For the number of images, regardless of the exam performed, no significant differences were observed in the number of images or fluoroscopy time, except for MCU (Table 4)

**TABLE 4:** Distribution of number of images, fluoroscopy time (min), PKA (Air kerma area product ‐mGy·m^2^), Ka,r (Air kerma at patient entrance reference point—mGy), according to weight category. Q1–Q3 (interquartile interval). MCU (micturating cystourethrograms), BM (barium meal), BE (barium enema), BS (barium swallow), MS (modified barium swallow), SBFT (small bowel follow‐through), FG (fluoroscopy genitography).

	N images				FT				PKA				Ka,r			
	*N*	median	(Q1–Q3)	*p* value	*N*	median	(Q1–Q3)	*p* value	*N*	median	(Q1–Q3)	*p* value	*N*	median	(Q1–Q3)	*p* value
**BE**																
<5	39	131	(56–309)	0.058	39	2	(1–3)	0.615	39	236	(140.5–351.9)	0.000	39	1.60	(1.0–2.9)	0.000
5 to <15	76	210	(79–435)		76	2	(1–3)		76	563	(302.4–1051.4)		76	2.75	(1.5–4.9)	
15 to <30	33	107	(44–170)		35	2	(1–3)		35	1698	(904.3–2856.0)		35	4.70	(2.6–7.7)	
30 to <50	21	219	(38–358)		22	3	(2–4)		22	3176	(2321.3–5603.9)		22	7.00	(4.2–13.1)	
50 to <80	8	54	(20–206)		8	2	(1–3)		8	7954	(2860.9–10 550.9)		8	19.55	(12.1–28.0)	
**MS**																
<5	8	850	(604–1432)	0.959	8	3	(2–4)	0.608	8	177	(117.1–260.3)	0.001	8	1.40	(0.8–1.5)	0.030
5 to <15	28	774	(462–1296)		29	3	(2–6)		29	732	(392.9–992.3)		29	2.30	(1.4–4.2)	
15 to <30	10	821	(418–1753)		12	4	(2–6)		12	1340	(541.9–2003.9)		12	3.00	(1.6–5.8)	
30 to <50	2	811	(322–1300)		2	3	(2–3)		2	2272	(790.8–3752.2)		2	5.60	(4.3–6.9)	
**BS**																
<5	31	384	(148–778)	0.497	31	2	(1–3)	0.686	31	211	(61.2–294.6)	0.000	31	1.10	(0.4–2.0)	0.001
5 to <15	27	456	(232–804)		28	2	(1–3)		28	394	(217.7–705.1)		28	1.40	(0.8–3.2)	
15 to <30	31	460	(247–1092)		33	3	(1–3)		33	670	(390.0–1037.2)		33	1.80	(1.4–3.0)	
30 to <50	21	390	(252–611)		22	2	(1–3)		22	1186	(788.1–2009.4)		22	3.30	(1.8–5.2)	
50 to <80	4	556	(286–1108)		4	2	(1–3)		4	1572	(945.3–4444.6)		4	2.95	(1.9–5.7)	
**FG**																
<5	3	591	(544–1109)	0.426	3	4	(2–4)	0.190	3	450	(67.6–486.2)	0.073	3	3.40	(1.5–4.5)	0.194
5 to <15	11	697	(524–1082)		11	4	(3–5)		11	1523	(626.3–1839.3)		11	8.90	(3.4–10.7)	
15 to <30	1	1494	(1494–1494)		1	10	(10–10)		1	2694	(2694.0–2694.0)		1	7.50	(7.5–7.5)	
30 to <50	1	673	(673–673)		1	7	(7–7)		1	22535	(22 534.8–22 534.8)		1	64.30	(64.3–64.3)	
**BM**																
<5	46	523	(303–1208)	0.071	46	3	(2–5)	0.077	46	299	(150.7–582.4)	0.000	46	2.20	(1.3–3.7)	0.000
5 to <15	94	506	(108–893)		95	3	(2–6)		95	678	(414.6–1.202.5)		95	3.10	(1.8–5.5)	
15 to <30	46	288	(65–528)		47	3	(1–4)		47	989	(477.7–1.714.9)		47	3.30	(1.6–6.7)	
30 to <50	27	409	(19–931)		27	4	(3–5)		27	2973	(1207.0–7487.9)		27	7.60	(3.7–12.7)	
50 to <80	1	717	(717–717)		1	6	(6–6)		1	23430	(23 429.7–23 429.7)		1	40.20	(40.2–40.2)	
**SBFT**																
<5	26	202	(92–557)	0.753	28	3	(2–4)	0.654	28	349	(185.1–780.3)	0.001	28	2.05	(1.4–5.1)	0.028
5 to <15	12	235	(66–861)		12	5	(1–6)		12	1666	(549.2–2447.5)		12	5.35	(2.9–9.3)	
15 to <30	2	556	(125–987)		2	2	(1–3)		2	1383	(451.5–2314.4)		2	3.30	(1.3–5.3)	
30 to <50	2	1075	(157–1993)		2	7	(2–11)		2	14651	(4475.4–24 827.2)		2	31.40	(8.0–54.8)	
**MCU**																
<5	68	439	(247–675)	0.000	71	3	(2–4)	0.010	71	173	(128.8–360.2)	0.000	71	1.60	(1.1–2.6)	0.000
5 to <15	88	366	(200–617)		89	3	(2–5)		89	715	(366.5–1419.0)		89	3.10	(1.6–5.1)	
15 to <30	64	320	(139–590)		65	3	(2–4)		65	1110	(591.7–2440.6)		65	3.00	(1.8–7.8)	
30 to <50	39	290	(127–546)		39	2	(2–4)		39	3312	(1403.1–6788.8)		39	7.00	(3.0–13.2)	
50 to <80	38	169	(23–323)		38	2	(1–3)		38	3655	(1564.5–6822.1)		38	5.90	(2.8–11.9)	
**IVU**																
<5	1	141	(141–141)	—	1	1	(1–1)	—	1	1405	(1405.1–1405.1)	—	1	4.60	(4.6–4.6)	—

In contrast, PKA and Ka,r showed statistically significant differences for all evaluated exams across the defined weight groups (*p* value < 0.05), except for genitography.

**FIGURE 2:**
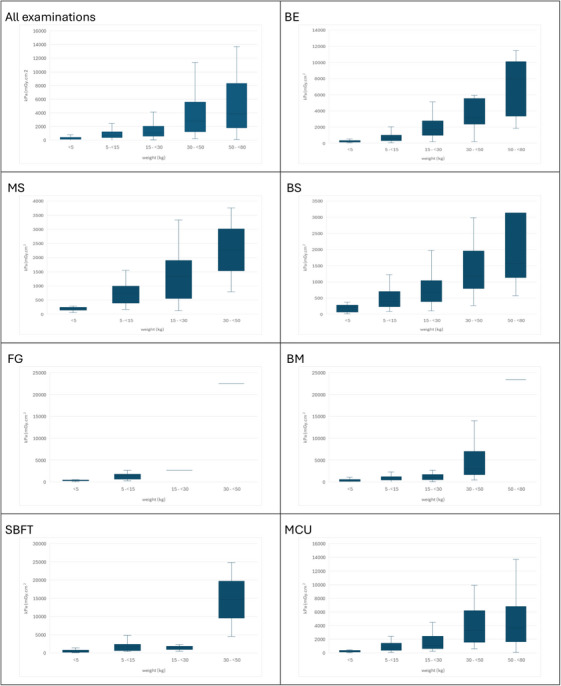
Boxplots showing the distribution of PKA values by weight category for each fluoroscopic examination: BS (barium swallow), BM (barium meal), MCU (micturating cystourethrogram), BE (barium enema), and MS (modified barium swallow).

This graphical representation (Figure 2) illustrates the visual component of the table reporting dose values, expressed in PKA, for patients stratified by weight categories across all fluoroscopic examinations evaluated. A progressive increase in dose values with increasing patient weight was observed in most examinations, along with differences in dose distributions among weight groups and across the different types of examinations.

**FIGURE 3:**
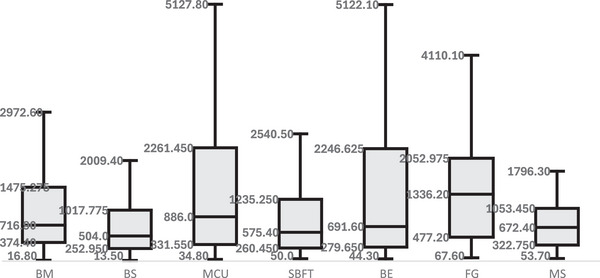
Box plot showing the distribution of PKA values by weight for each type of examination: BS (barium swallow), BM (barium meal), MCU (micturating cystourethrogram), BE (barium enema), MS (modified barium swallow).

Multiple box plot graphs (Figure 3) were generated using PKA dosimetric values for the different fluoroscopic examinations, revealing a wider dose distribution in MCU and BE, the two procedures with the highest representation of patients weighing over 50 kg. Sampling limitations were observed, especially among patients over 50 kg. For this weight group, BM, BS, and BE included only 1, 4, and 8 cases, respectively.

SBFT had sample sizes of *N* > 20 only for the 5 and 15 kg weight ranges. The MS met this requirement only for the 5 and 30 kg weight groups. FG did not reach the minimum of 20 participants in any weight category.

### 3.5 Specific clinical indications by type of examination

The main clinical indications for the requested examinations are described below:

- **MCU**: vesicoureteral reflux (VUR), neurogenic bladder and posterior urethral valve (PUV)
- **BM**: gastroesophageal reflux (GER), esophageal atresia, and esophageal stenosis
- **BE**: anorectal malformation (ARM), congenital megacolon (Hirschsprung's disease), and chronic constipation
- **BS**: esophageal atresia, esophageal stenosis, and dysphagia
- **MS**: functional and neurological dysphagia
- **SBFT**: gastroschisis, intestinal obstruction, and abdominal distension
- **FG**: anorectal malformation (ARM)

## 4 DISCUSSION

### 4.1 Overall contributions of the study

This study, a pioneering initiative in the Brazilian context regarding DRLs in pediatric fluoroscopy, provides a critical analysis of children's and adolescents' exposure to ionizing radiation. This study determined DRL values stratified by body weight, following the standard methodology recommended by the ICRP^7^ and EC.^8^ However, most international references stratified their results primarily by age and converted age groups into weight categories, as shown in Table 1.

Data were collected at a national tertiary referral center, with a specialized team and protocols adapted for the pediatric population. Nevertheless, the dosimetric values observed in several cases exceeded those reported in the international literature, including the European Diagnostic Reference Levels (EDRLs),^8^ the UK 2025 National DRLs (UKNRD)^20^ and other similar studies, such as those by Ashworth^4^ and Mbewe^21^ (Table 5).

**TABLE 5:** Comparison of local DRL values with international data, including examination parameters, KAP dose values (mGy·cm^2^), and fluoroscopy time (minutes). MCU (micturating cystourethrogram), BM (barium meal), BE (barium enema), BS (barium swallow), and MS (modified barium swallow).

Exam	*Weight group*	Weight median LDRL	PKA DRL median Mbewe^21^ Weight	FT LDRL median Weight	FT DRL median Mbewe^21^ Weight	UK NDRL 2025^20^	*Age group*	Age median LDRL	PKA DRL median Mbewe^21^ Age	EDRL^8^	PKA DRL P75 Ashworth^4^
BS	<5 5 to <15 15 to <30	211 394 670	76 199 432	2.2 2.1 2.6	2.4 2.8 2.9	200 400 500	*0 (<1 years)* *1 (1 to <5 years)* *5 (5 to <10 years)*	241 406 683	76 191 311		21 45
	30 to <50	1186	485	2.1	1.7	1800	*10(10 to <15 years)*	930	526		122
	50 to <80	1572		1.9		3000	*15(>15 years)*	1318	405		122
BM	<5 5 to <15 15 to <30	299 678 989	74 201 317	3.4 3.4 2.7	0.1 0.2 0.3	100 200 200	*0 (<1 years)* *1 (1 to <5 years)* *5 (5 to <10 years)*	384 807 992	74 156		9 18 58
	30 to <50	2973	2080	3.9	2.1	700	*10(10 to <15 years)*	2789	303		93
	50 to <80	23430		6.4		2000	*15(>15 years)*	4250	2080		132
MCU	<5 5 to <15 15 to <30	173 715 1110	114 157 438	2.7 3.1 2.6	3.6 2.6 3.2	100 300 300	*0 (<1 years)* *1 (1 to <5 years)* *5 (5 to <10 years)*	210 871 1330	151 273 665	300 700 800	9 12 23
	30 to <50	3312	449	2.4	1.8	400	*10(10 to <15 years)*	2587	306	750	
	50 to <80	3655		1.9		900	*15(>15 years)*	2080	1457		
BE	<5 5 to <15 15 to <30	236 563 1698	40 90	1.8 2.2 2	1.5 1.6		*0 (<1 years)* *1 (1 to <5 years)* *5 (5 to <10 years)*	307 714 1698	44 48		
	30 to <50	3176		2.7			*10(10* to* <15 years)*	2859	528		
	50 to <80	7954		1.8			*15(>15 years)*	11472	—		
MS	<5 5 to <15 15 to <30	177 732 1340	121 182 314	3.4 3.1 4.1	3 3 2.7		*0 (<1 years)* *1 (1 to <5 years)* *5 (5 to <10 years)*	343 797 542	132 163		
	30 to <50	2272		2.8			*10(10* to* <15 years)*	2757	418		
	50 to <80						*15(>15 years)*	3685	190		

The left side of the table presents the KAP values and fluoroscopy time categorized by weight ranges, comparing the local DRL (LDRL) values with data from Mbewe^21^ and the UKDRL 2025,^20^ the latter in which weight was estimated based on body thickness. The right side of the table shows the KAP values categorized by age ranges, including the local LDRL data as well as the corresponding values from Mbewe^21^ and EDRL.^8^ A possible explanation for these discrepancies lies in the previous lack of continuous and consistent verification of the radiation doses used in these examinations at our Institution, which hampers the implementation of optimization strategies. The comparative reference studies are primarily from Europe, where a culture of dose monitoring and optimization has been incorporated for decades, reflecting an institutionalized and ongoing concern with radiation exposure reduction.

Moreover, the profile of the population served in this study—predominantly composed of patients with rare diseases, congenital malformations, and genetic syndromes— implies higher technical complexity, including prolonged fluoroscopy time and consequently increased radiation doses. The comparison data originate from Europe and South Africa, where legislation allows pregnancy termination in cases of fetal abnormalities identified in utero,^25^, ^26^ potentially leading to a lower complexity of neonatal and pediatric pathologies in those regions.

The 2010 publication by the Royal College of Obstetricians and Gynaecologists^22^ identifies the main fetal abnormalities related to legally induced abortion as chromosomal disorders (e.g., Down and Edwards syndromes), central nervous system malformations, congenital heart defects, and genitourinary and musculoskeletal anomalies. Smith's 2011 study^2^ ^7^ reports that 79% of pregnancies with a prenatal diagnosis of congenital malformation are terminated in the United Kingdom, highlighting significant differences between the Brazilian and British populations.

As the first Brazilian study conducted in a public referral institution within the SUS to quantify radiation doses in pediatric fluoroscopy, the findings presented here can serve as a baseline for future updates and for the development of initiatives aimed at dose reduction.

### 4.2 Comparability of age‐ and weight‐based data

This study collected sufficient data to allow analysis by both weight and age categories. Body growth in childhood, with phases of physiological acceleration and deceleration exhibits a non‐linear, exponential pattern, whereas age progresses linearly.^25^ As a result, age‐based stratification does not accurately represent variations in body weight, composition, or thickness.

In Table 4, weight‐based LDRL values are, in almost all examinations, lower in the younger groups (<10 years) and show a more consistent progression aligned with physiological growth patterns.

In contrast, age‐based LDRL values display greater variability, with peaks and reversals in intermediate ranges, reflecting the weaker correlation between chronological age and actual body mass.

A similar pattern was observed in the data reported by Mbewe,^21^ who also analyzed dose values stratified by both weight and age. Their results demonstrated comparable inconsistencies between the two approaches.

Therefore, weight‐based grouping follows the physiological growth pattern and provides a more accurate reflection of dose progression, whereas age‐based grouping introduces inconsistencies, particularly in older children. Age‐based analysis using conversion tables is limited and may lead to inaccurate dose assessments, reinforcing international recommendations to define DRLs primarily by body weight, using age only when weight cannot be measured.

### 4.3 Considerations regarding rare exams and minimum criteria

In pediatrics, fluoroscopic exams are often limited to high‐specialization centers and performed infrequently. For MCU, the most common type of exam, the minimum N of 20 was met for all weight groups and all evaluated dose metrics (PKA, Ka,r, fluoroscopy time, and number of images), as well as for BM, BE, and BS—except in the >50 kg group for the latter three—thus allowing DRLs to be established fully in line with international recommendations.

ICRP Publication 135^7^ states that for high‐complexity procedures, in contexts with limited data or data from only a few centers, initial DRLs may be defined flexibly, even with fewer than 20 cases, as part of a dynamic process of DRL refinement. Based on this premise, we also defined DRLs for the remaining exam types and weight groups, even when the minimum number of 20 participants was not reached.

No DRLs were calculated for IVU, as only one case was recorded during the study period.

### 4.4 Discussion by examination type

In this study, the median dose values obtained at our institution (LDRL) were used for comparison with international DRL values, which are based on the 75th percentile (P75) of median doses derived from large multicenter datasets, such as those reported in the EDRL^8^ and UK NDRL.^20^ This approach is consistent with the recommended methodology for DRL evaluation, according to which an institution's median dose should fall below or near the reference level (P75) derived from broad population data, as it provides a more representative measure of local clinical practice—particularly in procedures with a limited number of examinations.

For single‐center studies (Ashworth,^4^ Mbewe^21^), comparisons should also be made between institutional medians, when available, since their P75 values reflect internal upper ranges rather than population‐based reference levels.

Table 5 presents a comparison of local DRL values, stratified by weight and age, with international multicenter data—such as the EDRL^8^ and UK NDRL^20^—and with single center studies, including the median values reported by Mbewe^21^ and the P75 values from Ashworth,^4^ as the medians in the latter were not fully disclosed.

The left side of the table shows the weight‐based data, including the local median values of PKA and fluoroscopy time, as well as the weight‐specific median PKA and FT values from Mbewe^21^ and the PKA data from the United Kingdom (UK NDRL^20^), which are based on body‐thickness measurements converted to weight according to the methodology described by Hart.^26^

The right side of the table presents the age‐based PKA values, including the local median LDRL data, the P75 values from EDRL,^8^ and those from Ashworth,^4^ which employ age‐to‐weight conversion tables.

In the weight‐stratified analysis, local values were found to be similar or slightly lower than the UK NDRL^20^ reference levels for the Barium Swallow (BS) examination, indicating good consistency with international standards. For the other examinations, however, the doses were higher, particularly in the higher weight ranges, which may reflect both the limited number of cases in these categories—reducing statistical representativeness—and technical or operational differences between protocols.

When compared with Mbewe,^21^ local doses were systematically higher across all examinations in weight as well as in age categories. The fluoroscopy time, however, remained similar to that reported by the author, except for the barium meal (BM) examination, in which the local time was substantially longer. This finding suggests that the observed discrepancies in dose are not solely attributable to exposure duration but may also be influenced by greater case complexity and variations in technical parameters.

In the age‐stratified analysis, Ashworth^4^ reported lower dose values across all comparable examinations, which may indicate the adoption of more optimized protocols tailored to a population with lower clinical complexity.

Regarding the EDRL, comparison could only be performed for the MCU examination age‐stratified, and it showed that local dose values were lower than the European standard in age groups below 5 years. However, in older age categories, a progressive increase in dose was observed, suggesting the need for further adjustments to technical and exposure protocols to enhance optimization.

### 4.5 Final considerations

The establishment of DRLs stratified by body weight reinforces the relevance of the present analysis, providing essential data for institutional self‐assessment and the development of dose optimization strategies. Furthermore, it allows the results to serve as a comparative reference for other imaging centers and national institutions, contributing significantly to the development of DRLs at both national and regional levels.

This is particularly relevant considering that, even in a center of excellence with a strong focus on pediatric radiological protection and a highly trained and committed technical team, the observed dose levels demonstrate the need for greater national engagement in implementing and promoting structured practices of dose monitoring and optimization.

The results indicate that technical expertise alone is insufficient to ensure effective dose control without established systems for monitoring and quality assurance. Such measures are fundamental to mitigating radiation exposure risks at the population level—especially in pediatric patients—and are more solidly established in the reference countries used in this study for comparison purposes.

### 4.6 Limitations of the study

The analysis was conducted using data from a single examination room, reflecting the practices of a single institution. This institution specializes in rare diseases, genetic disorders, and complex congenital malformations, which may lead to higher radiation doses due to the increased complexity of the studies. Therefore, the findings may not be generalizable to other centers or routine clinical settings.

The study also is limited by the small number of participants weighing over 50 kg and by examinations performed infrequently. These constraints may be mitigated by extending the data collection period, allowing for a more robust and representative dataset.

## 5 CONCLUSION

The results of this study highlight the importance of a rigorous methodological approach in defining DRLs for pediatric fluoroscopic examinations, especially in settings characterized by limited case numbers and high variability, such as single center studies.

The findings demonstrate that, although there is general conformity with international standards in the lower weight and age categories, there remains substantial potential for improvement in technical and exposure protocols—particularly in more complex examinations and higher weight categories.

This study presents the first systematized set of Brazilian data on local diagnostic reference levels (LDRLs) in pediatric diagnostic fluoroscopy, representing an initial milestone toward the standardization and optimization of radiological protection practices in the country.

Each institution can—and should—implement its own systematic program for collecting dose data stratified by weight, as this methodology provides more reliable and representative information on real clinical practice. Understanding the doses applied in each facility is essential, since these local datasets will eventually support aggregated national and regional databases, strengthening the culture of quality, radiological safety, and overall clinical performance.

## AUTHOR CONTRIBUTIONS

**Claudia M. C. Ribeiro**: Conceptualization; Methodology; Validation; Formal Analysis; Investigation; Resources; Data Curation; Writing—Original Draft; Writing—Review & Editing; Visualization; Supervision; Project Administration. **Tainá O. Chaves**: Conceptualization; Methodology; Validation; Formal Analysis; Resources; Data Curation; Writing—Original Draft; Writing—Review & Editing; Visualization; Project Administration. **Marcos Decnop**: Conceptualization; Methodology; Validation; Resources; Writing—Original Draft; Writing—Review & Editing; Visualization. **Renato Gonçalves de Mendonça**: Conceptualization; Methodology; Validation; Resources; Writing—Original Draft; Writing—Review & Editing; Visualization. **Margareth Catoia Varela**: Conceptualization; Methodology; Validation; Formal Analysis; Investigation; Resources; Data Curation; Visualization. **Cesar Augusto Viana de Araujo**: Conceptualization; Methodology; Validation; Investigation; Resources; Data Curation; Writing—Review & Editing; Visualization. **Saint Clair Gomes Junior**: Conceptualization; Methodology; Validation; Formal Analysis; Resources; Data Curation; Writing—Original Draft; Writing—Review & Editing; Visualization; Project Administration.

## CONFLICT OF INTEREST STATEMENT

The authors have no conflicts of interest to disclose.

## ETHICAL APPROVAL

All exams analyzed were performed on patients meeting justification criteria for imaging. This study was approved by the Research Ethics Committee of Fernandes Figueira Institute (CEP‐IFF), protocol CAAE: 63054222.0.0000.5269.

## Data Availability

The datasets generated and/or analyzed during the current study are not publicly available due to ethical and confidentiality restrictions.
