# Correction to “Local diagnostic reference levels for pediatric fluoroscopy in a Brazilian tertiary referral center: A weight‐based analysis”

**DOI:** 10.1002/acm2.70577

**Published:** 2026-05-10

Ribeiro CMC, ChavesTO, Decnop M, et al. Local diagnostic reference levels for pediatric fluoroscopy in a Brazilian tertiary referral center: A weight‐based analysis. J Appl Clin Med Phys. 2026;27:e70508. https://doi.org/10.1002/acm2.70508

Two issues were identified:

1. On page 3, the text appears as “018 years” and should be corrected to “0–18 years.”
2. In Tables 3 and 4, the dosimetric quantity (*P*_KA_) unit should be corrected to “mGy·cm^2^.” The current unit presentation (mGy·m^2^) may lead to the misinterpretation of the values reported in the tables and throughout the statistical analysis.

These corrections do not affect the study conclusions but are important to ensure the clarity and accuracy of the data presented.

We apologize for this error.

